# Vitamin D_3_ repletion versus placebo as adjunctive treatment of heart failure patient quality of life and hormonal indices: a randomized, double-blind, placebo-controlled trial

**DOI:** 10.1186/s12872-017-0707-y

**Published:** 2017-10-30

**Authors:** Heidi D. Moretti, Vincent J. Colucci, Bradley D. Berry

**Affiliations:** 1 Providence Saint Patrick Hospital, 500 W. Broadway St, Missoula, MT 59802 USA; 2 International Heart Institute of Montana, 500 W. Broadway, Missoula, MT 59802 USA; 3 University of Montana, 32 Campus Drive, Missoula, MT 59812 USA

**Keywords:** Vitamin D, Heart failure, B-type natriuretic peptide, Parathyroid hormone, Inflammation, C-reactive protein, Quality of life, 25-hydroxyvitamin D, Calcitriol

## Abstract

**Background:**

Vitamin D status may influence heart failure (HF) patient outcomes by affecting b-type natriuretic peptide (BNP), parathyroid hormone (PTH), and enhancing cardiac contractility. Vitamin D deficiency is associated with morbidity and mortality in HF patients. The objective of this study was to determine if vitamin D3 at a comparatively high dose would replete 25-hydroxyvitamin D (25(OH)D) stores, improve BNP, PTH, cardiopulmonary function, reduce inflammatory markers, and improve quality of life (QOL) in HF patients.

**Methods:**

This was a 6 month, parallel group, double-blind, placebo-controlled, single clinic center, randomized trial of supplemental vitamin D3 using a dose of 10,000 IU daily or placebo in 40 vitamin D deficient or insufficient (25(OH)D level ≤ 32 ng/ml) patients with stable New York Heart Association Class II-III HF in a specialty cardiology clinic. All variables were measured at baseline and 6 months. Values between the two treatment groups were assessed using Student’s t-test or Mann-Whitney Test. Univariate analysis of covariance was conducted to adjust for variance in baseline 25(OH)D.

**Results:**

All results were adjusted for baseline 25(OH)D. The change in BNP from baseline was ∆ +30 ± 950 pg/ml for treatment vs. placebo ∆ +400 ± 1900 pg/ml, *p* = 0.003. 25(OH)D serum levels rose by 49 ± 32 ng/ml in the treatment group vs 4 ± 10 ng/ml in the placebo group, *p* < 0.001. PTH and exercise chronotropic response index improved in the treatment group vs placebo group, respectively, but both were attenuated by adjustment ((∆-20 ± 20 pg/ml vs ∆ + 7 ± 53 pg/ml respectively (*p* = 0.01, adjusted *p* = 0.07)) and (∆ + 0.13 ± 0.26 vs. ∆-0.03 ± 02.9 respectively, *p* < 0.01, adjusted *p* = 0.17)). Other measured cardiopulmonary parameters remained unchanged. High sensitivity C-reactive protein (hsCRP) remained unchanged for women, but improved for men (∆-2 ± 4 treatment versus ∆2 ± 5 mg/L placebo, *p* = 0.05). QOL scores, including composite overall and clinical summary scores significantly improved in treatment compared to placebo (∆ + 10 ± 15 versus −6 ± 15, *p* < 0.01 and ∆ + 8 ± 14 versus −8 ± 18, *p* = 0.01, respectively).

**Conclusions:**

Repletion of 25(OH)D may improve QOL in HF patients and may help to normalize BNP, PTH, and hsCRP.

**Trial registration:**

Clinicaltrials.gov, Trial Registration Number: NCT01636570, First registered 3 July 2012.

**Electronic supplementary material:**

The online version of this article (10.1186/s12872-017-0707-y) contains supplementary material, which is available to authorized users.

## Background

Current lifetime risk for developing heart failure is estimated to be at 20–46% for people aged 45 and older in the United States [1], with a prevalence of the disease at approximately 23 million people worldwide [2]. Heart failure is a significant burden on the healthcare system, costing over 32 billion dollars per year with high risks of morbidity and mortality [3]. Data are accumulating to suggest that vitamin D, a secosteroid hormone, may modify disease course in numerous conditions, including heart failure [4–7]. Patients with both heart failure and vitamin D deficiency have an exceedingly high risk of morbidity and mortality, including sudden cardiac death [6, 8]. It is therefore important to assess if supplementation with the intent of achieving adequate circulating vitamin D levels also results in improved hormonal and inflammatory markers and patient health-related quality of life outcomes (QOL) in heart failure.

Vitamin D deficiency appears to be prevalent in subsets of patients with heart failure, including elderly patients at high northern latitudes or patients with concomitant kidney disease [8, 9]. Preliminary data suggests that vitamin D may improve ejection fraction (EF) [7, 10–12], and it appears that achieving a serum 25-hydroxyvitamin D (25(OH)D) level of greater than 40–50 ng/ml may be the threshold for benefit in heart failure patients [10]. Vitamin D may help resolve cases of hypocalcemic cardiomyopathy as shown in case reports of pediatric cardiomyopathy and heart failure related to rickets [13, 14]. This is a rare condition, therefore, extrapolating these data to the adult HF population would be inaccurate so inferences can’t necessarily be made for the adult heart failure population.

Results of vitamin D supplement trials are not consistent across all studies, but this is perhaps due to heterogeneity in study design, dosing regimens, and biological flaws [15, 16]. These biological flaws may be overlooked when evaluating the research for vitamin D. Further, biological flaws are created when the design does not include evaluation vitamin D status, use pulse dosing, or the dose of vitamin D is not sufficient to change serum vitamin D status. Frequently, these flaws can lead to null results [14–16].

There is also a gap in the literature related to vitamin D and functional outcomes such as health-related QOL and exercise cardiopulmonary status (CPX) in heart failure patients. QOL and CPX are strong prognostic markers of morbidity and mortality [17, 18]. Vitamin D is directly involved in the calcium-related contractility in the heart. Cardiomyocytes and vascular smooth muscle cells possess both vitamin D receptors and calcitriol-dependent calcium binding proteins [19, 20]. The presence of both vitamin D receptor and 1,25-dihydroxyvitamin D[1,25(OH)2D]calcitriol-dependent calcium binding proteins in these tissues allows for the onsite conversion of 25(OH)D to calcitriol 1,25(OH)2D and calcium-dependent cellular processes, underscoring the requirement of vitamin D in these tissues. Depletion of vitamin D3 in the rat model results in impaired contractile function as well. [21]. Therefore, the aim of the current study is unique as we sought to determine if vitamin D3 in higher doses would result in sufficient circulating 25-hydroxyvitamin D levels, and subsequently result in changes in surrogate markers of heart failure outcomes.

## Methods

We aimed to evaluate the effects of vitamin D on hormonal markers, functional outcomes, and QOL in our center. Therefore, we hypothesized that vitamin D3 at 10,000 IU daily would be sufficient to replete 25(OH)D stores over a 6-month period, and reduce or normalize b-type natriuretic peptide (BNP), our primary outcome measure. Our secondary outcome measures included changes in or improvement of parathyroid hormone (PTH), high sensitivity c-reactive protein (hsCRP), QOL, and cardiopulmonary function using CPX.

### Study design

This was a single center, parallel group, randomized, controlled, double–blinded prospective trial. Enrollment and all assessment were conducted at a specialty heart failure clinic by cardiologists, in Western Montana between fall of 2012 and spring of 2015. Clinic patients with New York Heart Association (NYHA) Class II or III heart failure were eligible for inclusion into the study if they were 18 years or older, had been on stable and guideline directed medical therapy (GDMT) for greater than 3 months, and had a 25(OH)D level of ≤32 ng/ml. Patients who were pregnant, breastfeeding, or with any clinically unstable medical disorder were excluded. All data, including QOL, laboratory values, and cardiopulmonary function were collected from the clinic by research coordinators and nursing staff. Patients currently taking ≤1000 IU vitamin D daily could enroll in the trial. Randomization was computerized in a permuted block size of 4 and occurred off-site at a university pharmacy department. Vitamin D3 and placebo bottles were given sequentially numbered identical containers and administered serially. The trialists, patients, and caregivers were blinded to the randomization until all data were collected and collated. Patients were excluded from the trial if they presented with any of the following conditions: hypercalcemia, nephrolithiasis, sarcoidosis, acute decompensation of heart failure, and/or any clinically unstable systemic medical disorder, including severe pulmonary disease. Vitamin D3 has been shown to be safe long-term at doses of 10,000 IU per day [22]. Vitamin D dose at a given amount seems to result in a plateau in 25 (OH)D at or around 1 month, so we estimated that 6 months of dosing would be sufficient to have an effect on hormonal and cardiopulmonary status [22]. All patients provided written, informed consent and the trial was approved by the Providence Saint Patrick Hospital Institutional Review Board. Patient variables were divided into gender differences for men and women. Vitamin D3 at 10,000 IU per day (Biotech Pharmacal, Fayetteville, AR) or identical placebo (dextrose) was given in parallel design. Patients remained on all current medications as prescribed, and changes in any baseline medication dosages or forms were recorded. All patients enrolled in the study were receiving best tolerated and GDMT, which included Angiotensin Converting Enzyme Inhibitor (ACE), Angiotensin Receptor Blocker (ARB), beta blockers (Table 1), and aldosterone antagonists. They were also receiving statins and loop and/or non-loop diuretics as indicated. All medications had been stable for at least 3 months before enrollment.

**Table 1:** Baseline Demographics

	All Groups	Placebo	Treatment	*P*-value
Age (years)	67 ± 14	65 ± 16	67 ± 11	0.53
Gender (n, % Female)	13/40 (33%)	6/20 (30%)	7/20 (35%)	0.74
Race (n, % Caucasian)	38/40 (95%)	19/20 (95%)	19/20(95%)	1
BMI (kg/m^2−^)	29 ± 5	28 ± 5	31 ± 5	0.33
HR (BPM)	72 ± 11	70 ± 10	74 ± 11	0.92
Systolic BP (mmHg)	113 ± 16	117 ± 19	110 ± 14	0.38
Diastolic BP (mmHg)	69 ± 10	71 ± 10	67 ± 10	0.42
EF (%)	28 ± 9	30 ± 11	27 ± 7	0.13
Ischemic (#, %)	25/40 (63%)	14/20 (70%)	11/20 (55%)	0.33
Nonischemic (#, %)	15/40 (37%)	6/20(30%)	9/20 (45%)	0.33
ACE/ARB (#, %)	33/40 (83%)	17/20 (85%)	16/20 (80%)	0.68
Digoxin (#, %)	4/40 (10%)	2/20 (10%)	2/20 (10%)	1
Beta Blocker (#, %)	32/40 (80%)	14/20 (70%)	18/20 (90%)	0.11
Loop Diuretic (#, %)	28/40 (70%)	13/20 (65%)	15/20 (75%)	0.49
Spironolactone and/or Eplerenone	31/40 (78%)	16/20 (80%)	15/20 (75%)	0.70
Statins (#, %)	29/40 (73%)	15/20 (75%)	14/20 (70%)	0.72

Data are expressed as mean ± standard deviation (SD) or number and percentages. *Abbreviations*: Body Mass Index (*BMI*), Heart Rate (*HR*), Blood Pressure (*BP*), Ejection Fraction (*EF*) Angiotensin Converting Enzyme Inhibitor (*ACE*), Angiotensin Receptor Blocker (*ARB*)

Patients were evaluated at baseline and at 6 months for all clinical and laboratory data, and they had complete medical assessments, QOL, and CPX tests at both visits. At 3 months, both 25(OH)D and serum calcium levels were drawn. Due to the high number of patients screened taking vitamin D at 1000 IU daily that remained deficient in 25(OH)D, we modified the protocol to include patients who were taking up to 2000 IU per day of vitamin D prior to enrollment that had baseline levels of 25(OH)D ≤ 32 ng/ml. If 25-hydroxyvitamin D levels exceeded 150 ng/ml or calcium level exceeded 10.5 mg/dl at 3 months, vitamin D3 dose was decreased to 5000 IU per day. If any adverse effects were observed by the patient or cardiologist, the patient was withdrawn from the trial.

### Laboratory parameters

BNP, PTH, hsCRP, and 25(OH)D, hsCRP, complete metabolic panels and complete blood counts were all measured using commercially available kits.

### QOL

The Kansas City Cardiomyopathy (KCCQ), a validated tool that is both sensitive and specific for assessment of change in health-related QOL, was interviewer administered at baseline and at 6 months of treatment [23]. Briefly, the KCCQ has domains of physical function, symptom frequency and severity, social function and QOL; composite scores are derived from these domains. Scores are computed for a range of 0–100 for each domain, in which higher scores reflect better health status.

### CPX

All subjects, following 2 min of rest, completed an initial 1-min, constant-rate stair step. After the initial workload, speed and grade were incrementally increased at 1-min intervals to peak attained exertion. Ventilatory expired gas analysis was collected at rest and throughout exercise using a miniaturized metabolic cart (Shape HF cardiopulmonary testing system, St. Paul, MN). The Shape analyzer was self-calibrated daily using an auto-cal performance test. Heart rhythm was assessed on a 4-lead electrocardiogram, and arterial O_2_ saturation was monitored with finger pulse-oximetry (Envitec D-23966, Wismar, Germany).

Minute ventilation, the end-tidal partial pressures of O_2_ (PETO_2_) and CO_2_ (PETCO_2_), oxygen consumption (VO_2_), and carbon dioxide production (VCO_2_) was acquired breath by breath and averaged over 30-s intervals every minute until exhaustion (progressive exercise to peak oxygen consumption, VO_2_peak). The data points between the averaged value during the first 30 s of the initial work load and at peak exercise was used to calculate the ventilator efficiency (V_E_) to VCO_2_ slope and changes in partial pressure end-tidal CO_2_. In addition, the chronotropic response index (CRI) and O_2_ uptake efficiency slopes were determined during exercise as previously described [24]. Greater values of oxygen uptake efficiency slope indicate a higher ventilatory efficiency, and a larger CRI slope is an indicator of improved heart rate reserve.

### Statistical analysis

We set a threshold of 90% power to detect a between-group difference in BNP (based on 74 ± 25 pg/ml between-group differences seen in the literature) to determine a need for at least 6 patients in each [25]. The differences between the means of the two data sets were assessed by 2-sided independent samples *t* test. For data not normally distributed, the analysis was performed by means of Wilcoxon signed-rank test. A *p*-value of 0.05 or less was considered statistically significant. For the study to have >90% power to detect a between-group difference for our primary outcome measure of BNP of 74 pg/ml with SD value of 25 mg/ml found in the literature, the number of patients in the participating group must be 6 or more [25]. A two-tailed z-value related to *α* = 0.05and a two-tailed z-value related to *β* = 0.1 were used for the estimate together with the expectation that vitamin D_3_ supplementation would lead to a reduction in BNP.

We increased our goal recruitment size to 20 per group to allow for gender differences to be assessed. Data presented was analyzed using SPSS version 23 (IBM® Corporation, Armonk, New York), and expressed as the mean ± standard deviation (SD). Normality was assessed for each variable by the Shapiro Wilk test. Values between the two treatment groups were assessed using Student’s t-test or Mann-Whitney Test for normally distributed and non-normally distributed data, respectively. Univariate analysis of covariance was conducted to adjust for variance in baseline vitamin D status. Significance was set a priori at α = 0.05. Other continuous variables (expressed as means, SD) and categorical variables (expressed as frequencies) were collected during the study to characterize the group. Subgroup analysis was conducted for gender differences for all outcome variables. Differences in race could not be conducted due to the patient demographic of the region was primarily white. Data were analyzed per protocol analysis. All data is available in the attached data repository (Additional file 1). The CONSORT checklist is attached in Additional file 2.

## Results

**Fig. 1:**
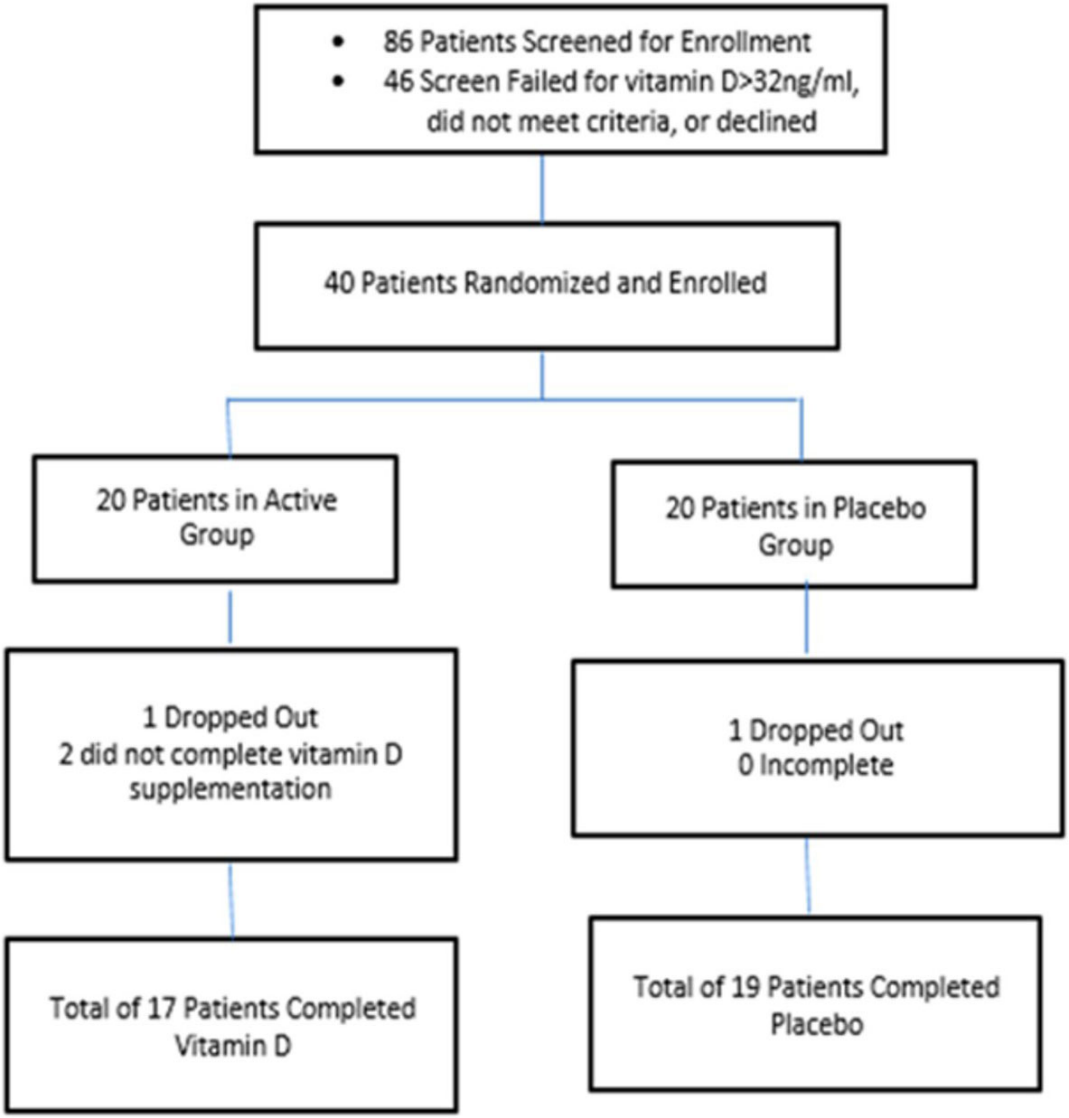
Consort Diagram. Study Design and Enrollment

A total of 40 patients were enrolled into the study (Fig. 1). Of these, 2 dropped out for unrelated reasons (1 treatment, 1 placebo), and 2 patients did not complete the vitamin D3 supplementation (both in the treatment group, not related to intolerance, but related to compliance). A total of 17 pts. had completed the study in the treatment group, and 19 in the placebo group. Baseline characteristics were similar between both groups for all parameters measured (see Table 1). Additionally, all laboratory parameters were similar between the groups at baseline, with the exception of PTH (*p* = 0.002, Table 2). All patients tolerated the vitamin D3 treatment or placebo, and no patients were required to have a reduction in dosage or discontinuation of treatment.

**Table 2:** Baseline and Post-Treatment/Placebo Laboratory Parameters

	Placebo Baseline	Placebo 6 months	Treatment Baseline	Treatment 6 months	*p*-value Baseline	*p*-value 6 months
Sodium (mEq/L)	140 ± 2	140 ± 3	138 ± 3	139 ± 2	0.11	0.24
Chloride	104 ± 4	104 ± 3	99 ± 21	104 ± 3	0.25	0.25
Co2	26.3 ± 2.9	25.8 ± 2.1	25.0 ± 3.0	25.5 ± 3.0	0.46	0.86
Potassium(mEq/L)	4.1 ± 0.8	4.1 ± 0.3	4.4 ± 0.3	4.3 ± 0.4	0.29	0.15
BUN (mg/dl)	22 ± 12	23 ± 13	24 ± 10	27 ± 13	0.46	0.33
Creatinine	1.3 ± 0.6	1.3 ± 0.6	1.2 ± 0.4	1.3 ± 0.5	0.29	0.86
Glucose (mg/dl)	115 ± 41	105 ± 36	118 ± 35	108 ± 23	0.64	0.79
Calcium (mg/dl)	8.9 ± 0.5	8.9 ± 0.8	9.0 ± 0.4	9.2 ± 0.4	0.71	0.18
Hemoglobin (g/dl)	14.1 ± 1.9	13.9 ± 1.5	14 ± 6.2	13.4 ± 1.5	0.63	0.51
Hematocrit (%)	44.5 ± 12.8	41.5 ± 4.4	42.6 ± 11.0	40 ± 4.6	0.39	0.31
BNP (pg/ml)	1800 ± 400	2300 ± 950	1600 ± 550	1400 ± 600	0.43	0.31
PTH (pg/ml)	90 ± 96*	93 ± 112	58 ± 28	45 ± 19	0.002	0.002
hsCRP (mg/ml)	3.5 ± 3.1	4.4 ± 3.9	5.4 ± 5.5	3.8 ± 1.1	0.42	0.18
25(OH)D (ng/ml)	20 ± 7	20 ± 9	19 ± 7	70 ± 28	0.8	0.001

Data are expressed as mean absolute values + standard deviation (SD). *Abbreviations*: Blood Urea Nitrogen (*BUN*), B-type Natriuretic Peptide (*BNP*), Parathyroid Hormone (*PTH*), high-sensitivity c-reactive protein (*hsCRP*), and 25-hydroxyvitamin D (*25(OH)D*)

*Indicates significant difference in PTH between placebo and treatment group at baseline, p=0.002

**Fig. 2:**
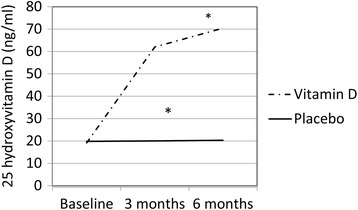
Change in 25 hydroxyvitamin D (25(OH)D) level and calcium level at 3 and 6 months. 25(OH)D significantly increased from baseline in the treatment group by 3 months and 6 months compared to placebo group, *p = 0.001. Calcium levels remain unchanged

At 3 months, calcium levels were unchanged from baseline in both groups (8.9 ± 0.6 mg/dl in the placebo group and 9.0 ± 0.3 mg/dl in the treatment group (Fig. 2). At 3 months, 25(OH)D levels reached an average of 60 ± 30 ng/ml in the treatment group and remained at 20 ng/ml in the placebo group (*p*-value between groups of <0.001). By 6 months, vitamin D status rose to 70 ± 28 ng/ml in the treatment group while the placebo group remained unchanged (20 ± 7 ng/ml).

**Fig. 3:**
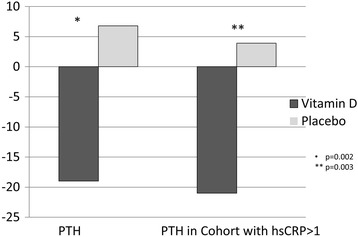
Change in PTH from baseline at 12 weeks and 6 months. Change in PTH is depicted on the left, and change in PTH in cohort with hsCRP > 1 is shown in the left columns. * indicates *p* < 0.05 and ** indicates *p* < 0.01

All results were adjusted for baseline 25(OH)D. In a total of 17 patients, BNP improved in the treatment group, ∆ + 30 ± 950 pg/ml, compared to the placebo group of 19 patients, ∆ + 400 ± 1900 pg/ml (*p* = 0.002) (Table 3). After adjustment for baseline 25(OH)D, the difference between groups for BNP remained significant (*p* = 0.003). Serum PTH also significantly improved (placebo ∆ + 7 ± 53 pg/ml versus treatment ∆-20 ± 20 pg/ml, p = 0.002, Fig. 3). This difference was attenuated when adjusted for baseline vitamin D (*p* = 0.07). Absolute scores for PTH were 90 ± 96 pg/ml at baseline and 93 ± 112 pg/ml at 6 months for the placebo group versus 58 ± 28 pg/ml at baseline and 45 ± 19 pg/ml at 6 months for the treatment group (Table 2). Adjustment for baseline 25(OH)D attenuated the difference between the groups (p = 0.07). hsCRP levels were not significantly different between the groups.

**Table 3:** Changes in lab parameters and KCCQ

	Vitamin D	95% CI	Placebo	95% CI	*p*-value	Corrected model *p*-value
BNP	30 ± 950	−10, 70	400 ± 1900	210–600	0.002*	0.003*
PTH	−20 ± 20	−28, −11	7 ± 53	16, 30	<0.002*	0.07
hsCRP	−1 ± 5	−3, 1	1 ± 6	−2, 4	0.18	0.21
25-(OH)D	51 ± 32	39,62	1 ± 6	−2, 3	<0.001*	Na
Physical Score	9 ± 14	3,15	−5 ± 20	−4, 14	0.02*	0.07
Symptom Frequency	10 ± 28	−2, 28	−6 ± 17	−2, 13	0.03*	0.06
Symptom Burden	12 ± 20	3, 20	−7 ± 15	−14, 0	0.002*	0.05*
Total Symptom	11 ± 22	1, 22	−6 ± 17	−13, 1	0.013*	0.03*
Stability Score	3 ± 35	−13, 18	1 ± 14	−5,7	0.88	0.84
QOL Score	11 ± 21	3, 21	−2 ± 20	−11, 7	0.05*	0.11
Social Score	10 ± 24	0, 24	−4 ± 27	−16, 8	0.14	0.27
Overall Summary Score	10 ± 15	4,17	−6 ± 15	−13, 1	<0.02*	0.01*
Clinical Summary Score	8 ± 14	2, 14	−8 ± 18	−16, 0	0.02*	0.01*

Data are expressed as mean ± SD, 95% CI

*Abbreviations*: Kansas City Cardiomyopathy Questionnaire (*KCCQ*), Standard Deviation (*SD*), Confidence Interval (*CI*), B-type Natriuretic Peptide (*BNP*), Parathyroid Hormone (*PTH*), high sensitivity c-reactive protein (*hsCRP*), 25 hydroxyvitamin D (*25-(OH)D*), Quality of Life Score (*QOL*)

*Indicates significant difference between groups

To assess in an exploratory way if inflammation affected PTH response to vitamin D, a cohort of the patients with hsCRP levels greater than 1 were analyzed as well. In this cohort, PTH was significantly improved by vitamin D ∆-21 ± 20 compared to the placebo group ∆4 ± 17 pg/ml, *p* = 0.005 (Fig. 3).

Parameters from CPX test remained unchanged except for chronotropic response index (CRI) (Table 4), which significantly improved at 6 months in the vitamin D3 group (∆0.1 ± 2.4 treatment versus −0 ± 2.9 placebo, *p* = 0.009). This was attenuated after adjustment for baseline vitamin D (*p* = 0.17).

**Table 4:** Changes in Cardiopulmonary Exercise Tests

	Vitamin D	Placebo	*p*-value	Corrected model *p*-value	Vitamin D CI (lower)	Vitamin D CI (higher)
Peak VO_2_ ml/kg/min	−1.7 ± 3.4	0.4 ± 3.5	0.12	0.30	−3.2	−0.21
Peak METS	−0.5 ± 1.1	−0.2 ± 0.7	0.43	0.32	−0.9	0.03
Peak Tidal Vol	30 ± 380	−40 ± 240	0.55	0.72	−132.5	195.3
CRI	0.1 ± 0.20	0 ± 2.9	0.009*	0.17	0.02	0.24
V_E_ Slope	3.0 ± 6.7	0.8 ± 5.7	0.36	0.51	0.1	5.9
Heart Composite	0.3 ± 0.7	0.1 ± 0.6	0.32	0.57	0.28	0.36
Pulmonary Vascular Score	0.1 ± 0.5	0.1 ± 0.6	0.97	0.34	−0.1	0.4
Deconditioning Score	0.3 ± 0.6	0.2 ± 0.7	0.65	0.19	0.03	0.53
PETCO2(mmHg)	−1.6 ± 3.4	−0.6 ± 1.9	0.30	0.40	−3.1	−0.11
Shape Score	0.1 ± 1.6	0.3 ± 1.0	0.65	0.71	−0.6	0.8
Peak RER	0.1 ± 0.3	−0 ± 0.1	0.26	0.44	0	0.24
OUES	−0.1 ± 0.4	−0.3 ± 0.5	0.12	0.32	−0.14	0.36

Data are expressed as Δ ± SD, 95% CI)

*Abbreviations*: Standard Deviation (*SD*), Confidence Interval (*CI*), Volume of oxygen consumed (*VO*_*2*_), metabolic equivalents (*METS*), volume (*Vol*), Ventilatory Efficiency (*V*_*E*_), Respiratory Exchange Ratio (*RER*), Oxygen Uptake Efficiency Slope (*OUES*)

^*^Using Mann-Whitney

To determine within-gender differences, data were analyzed for all the outcome measures. Changes between groups for BNP, cardiopulmonary function, and QOL remained similar to the composite group for most outcomes with the exception of hsCRP; for men, hsCRP significantly improved (hsCRP Δ −2 ± 4 versus 2 ± 5 mg/L for the placebo group, *p* = 0.05), but not for women (∆1 ± 5 treatment versus 0 ± 4 for placebo, *p* = 0.87).

**Fig. 4:**
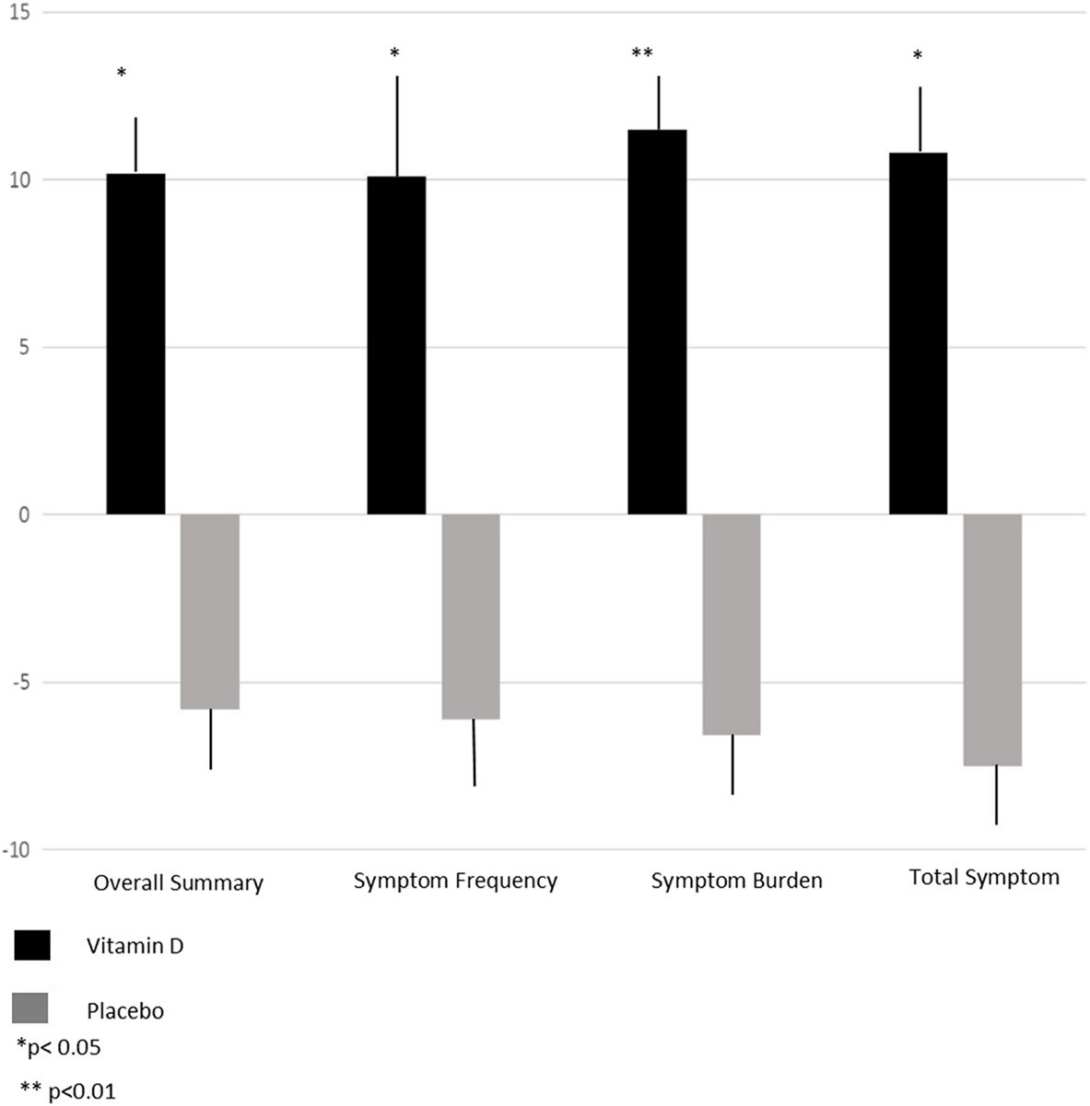
Change in Quality of Life Scores using the Kansas City Cardiomyopathy Questionnaire

KCCQ scores significantly improved compared to placebo in the domain of physical (∆ + 9 ± 14 treatment versus ∆-5 ± 20 placebo), QOL (∆ + 11 ± 21 treatment versus ∆-2 ± 19 placebo), and for both the overall summary (∆ + 10 ± 15 treatment versus ∆-6 ± 15 placebo) (Fig. 4), and the clinical summary (∆ + 9 ± 14 treatment versus ∆-8 ± 18 placebo) (Table 3). Stability scores and social scores did not differ significantly between the groups. Symptom frequency scores trended towards improvement in the vitamin D group, and significantly improved for the vitamin D group for symptom burden (*p* = 0.002) (Fig. 4). Adjustment for baseline vitamin D status slightly attenuated all scores except the overall and clinical summary scores (Table 3).

## Discussion

The conclusions from our research support prior findings that vitamin D3 supplementation at pharmacological doses may benefit patients with heart failure (7,10–12). However, clinical trials using vitamin D supplementation with hard endpoints such as cardiovascular death, hospitalizations, or all-cause mortality still have not yet been conducted. So far, vitamin D supplementation guidelines have not been included in heart failure guidelines by European Society of Cardiology or American College of Cardiology (26–27). Adequate research of vitamin D measuring sufficient repletion in a large number of patients also has not been performed.

### BNP findings

Our findings demonstrate that therapeutic vitamin D3 treatment is significantly better than placebo for BNP measures when adjusted for baseline vitamin D in patients with reduced ejection fraction heart failure. These findings are noteworthy because BNP has been shown to be strongly associated with recurrent hospitalization and mortality in this patient population. BNP was the most powerful independent marker of outcome in heart failure patients in the Valsartan Heart Failure study [26]. A limitation to our current finding is that BNP variability between patients was large, and our enrollment criteria did not specify a range for BNP.

Prior research is conflicting related to vitamin D and its influences on BNP. A study by Jiang et al. failed to show a change in BNP after vitamin D supplementation, but a more recent supplement trial found that vitamin D dramatically improved BNP levels using 200,000 IU vitamin D per week [27, 28]. However, a limitation of this trial was a lack of randomization [28]. In the meta-analysis failing to find a change in BNP, biological flaws were present, as the average 25(OH)D level was not taken into consideration. Our current study is the highest dose per day of any study to our knowledge. Mechanisms of vitamin D3 actions related to BNP have not been fully described, but the vitamin D receptor likely plays a role in regulating the BNP gene promoter [29].

### QOL

In our study, QOL scores improved significantly in most domains, and this remained robust after adjustment. Our study is the first to provide vitamin D3 at a dose shown to replenish vitamin D stores, and also measure QOL. Prior research demonstrates that improvement in QOL predicts event-free survival in heart failure patients [30]. Comparatively, current treatments don’t approach the effect size for QOL that our study has shown. For example, the Angiotensin-Neprilysin Inhibition (LCZ696) versus Enalapril trial [31] demonstrated a 2.99-point worsening in KCCQ clinical summary with the use of LCZ696, which was statistically better than conventional therapy with Enalapril (worsened KCCQ by 4.63 points). In contrast, our KCCQ clinical summary had an 8 point average improvement. The implications of this could be large if replicated.

Heart failure results in diminished QOL, in part due to frailty; vitamin D3 may help by preserving and improving lean body mass and strength [32–34]. A recent trial in heart failure patients failed to show improvements in physical function and QOL in heart failure patients. However, serum 25(OH)D never surpassed 32 ng/ml on average [34]. Two pulse doses of vitamin D2 is likely insufficient to overcome a deficiency in heart failure [22]. In contrast, when 200,000 IU vitamin D3 per week was given to heart failure patients, the 6-min walk test was significantly improved from baseline [28].

### CPX

While most CPX remained unchanged in our study, we did find that vitamin D seemed to improve CRI. However, adjustment for baseline vitamin D attenuated this. Our study may not have been long enough in duration to find differences in cardiopulmonary exercise testing as a whole. Our finding that chronotropic response may be improved by vitamin D deserves further attention since this index is inversely associated with mortality in heart failure patients [35].

Treatment of heart failure with Angiotensin Converting Enzyme Inhibitors and Angiotensin Receptor Blockers has been documented to improve cardiopulmonary function and exercise capacity [36]. The mechanism of action of these medications is inhibition of the Renin-Angiotensin-Aldosterone System (RAAS), in particular, angiotensin II. Vitamin D analogs block renin upregulation and ANGII accumulation in animals [37]. Vitamin D3 thus may also improve CPX with synergistic effects of established treatments. Further research with vitamin D3 and vitamin D cofactors with longer duration may further elucidate the effects of vitamin D3 on CPX.

### PTH

PTH was reduced by 20 pg/ml in our vitamin D3 group; other studies have had similar findings in heart failure to a lesser magnitude [38]. Adjustment for baseline 25(OHD status in our study did attenuate this significance; however, PTH and 25(OH)D are quite inter-related, so this does not appear to minimize our treatment effect. However, our baseline PTH levels were higher than most prior trials [39]. Vitamin D3, therefore, may be more beneficial in patients with markedly elevated PTH. Our groups were not matched at baseline in regard to PTH. This is possibly true because our enrollment criteria did not include PTH parameters for exclusion or inclusion. Serum calcium also didn’t vary between the groups, but we did not allow patients with hypercalcemia into the study for safety concerns. This may explain why the baseline calcium levels looked similar between groups. However, with no change for PTH in the placebo group after 6 months, our reduction of PTH in the vitamin D group does appear to be related to vitamin D repletion. National Health and Nutrition Examination Survey (NHANES) data have demonstrated that PTH is associated with CRP [40]. We found that PTH also was associated with hsCRP, and selecting for patients with hsCRP greater than 1 further strengthened the effect of vitamin D3 on PTH.

Vitamin D directly affects the parathyroid gland, and deficiency of vitamin D results in secondary hyperparathyroidism. Hyperparathyroidism is strongly associated with HF outcomes, with elevated PTH directly related to both hospitalizations and mortality [41, 42]. Additionally, PTH levels in heart failure patients are elevated compared to healthy adults [43], suggesting that PTH dysregulation is a component in the complex pathology of heart failure. Optimization of PTH does not occur until 25(OH)D surpasses 40 ng/ ml [44].

### Vitamin D status

Our results indicate that a vitamin D3 supplement of 10,000 IU per day effectively corrects vitamin D deficiency in heart failure without evidence of adverse effects. Our dose-response is like other studies that have used 10,000 IU of vitamin D3 daily, and a steady state of serum 25(OH)D was reached by month 3 [22]. Further research is needed to evaluate if vitamin D dosing guided by 25(OH)D levels would be equally effective or superior, or if additional nutrient cofactors would further improve patients’ response [15, 16].

Vitamin D deficiency increases the risk of all-cause mortality, incident cardiovascular disease, and sudden cardiac death in the general population [45]. Patients with heart failure and vitamin D deficiency have an almost 3-fold risk of death and 5-fold risk of sudden cardiac death compared with those who are vitamin D replete [6]. Vitamin D3 affects genetic expression related to all cardiovascular cell types; it thereby helps regulate cellular differentiation, apoptosis, hormone production, and oxidative stress [46, 47]. The efficacy of vitamin D3 is related to its ability to provide enough substrate, so the translation of vitamin D studies should involve serial measurements of vitamin D status. Vitamin D repletion is an appealing target therapy due to its numerous physiological benefits for humans [15, 16].

Vitamin D activated to 1,25(OH)2D calcitriol is considered a pleiotropic hormone, and vitamin D receptors occupy 2776 genome positions. Vitamin D directly affects the expression of many genes, and a recent study showed that improvement in vitamin D status after supplementation results in correction of genetic expression [47]. In this study, there was a 1.5 fold change in the expression of 291 genes with vitamin D supplementation in vitamin D deficient healthy adults; 66 of those genes had dramatic changes in expression [47]. Tripartite Motive Containing 27 gene (TRIM27) and Early Growth Response (EGR-1) gene among many others were down-regulated; TRIM27 gene is associated with oxidative stress, immunoglobin E mediated immunity among others, and EGR-1 is linked to vascular health.

A recent meta-analysis demonstrated that supplemental vitamin D3 increases circulating 1,25(OH)2D levels significantly, and its actions are mediated through vitamin D receptors [48]. This is an important finding because circulating 1,25(OH)2D levels are associated with better outcomes in end stage HF [49]. Cardiomyocytes, immune cells, endocrine, and vascular smooth muscle cells have vitamin D receptors and outcomes in HF are associated with circulating calcitriol levels [50–52]. These receptors throughout the body also allow for onsite conversion of1,25(OH)2D calcitriol and calcium-dependent cellular processes, and high concentrations of calcitriol in these cell types are likely necessary to trigger gene expression [51]. These key effects of vitamin D on post-translation and non-genomic actions, such as transmembrane calcium transport, provide insight into the beneficial mechanistic action of vitamin D on the cardiovascular system [53].

Vitamin D deficiency, as defined by the Institute of Medicine is <20 ng/ml for 25(OH)D levels, while insufficiency is generally considered to be 20–30 ng/ml [54]. New research suggests, however, that a circulating level of 20 ng/ml presents a 2-fold risk of secondary hyperparathyroidism over levels greater than 30 ng/ml [55]. Elevated PTH is associated with poor outcome in HF patients [56]; our findings demonstrate that vitamin D3 supplementation can markedly improve PTH concentrations at higher circulating levels of 25(OH)D, further substantiating the existing data showing that higher circulating 25(OH)D concentrations help mitigate hyperparathyroidism. Vitamin D status is closely related to PTH status and 25(OH)D appears to suppress PTH across at all levels of 25(OH)D status [57]. Additionally, it has become clear that the extraskeletal benefits of vitamin D occur at higher circulating blood levels of 25(OH)D; for example, respiratory infection risks are reduced for those individuals with 25(OH)D in the range of 50 ng/ml or more [54]. For these reasons, we chose a higher cutoff criterion for enrollment than Institute of Medicine guidelines.

A question remains as to whether heart failure patients require vitamin D3 treatment only in the cases of 25(OH)D < 20–30 ng/ml, or if all patients with heart failure would benefit from supplementation despite normal circulating 25(OH)D levels. As of yet, evaluation of vitamin D status is not routinely performed in heart failure. However, patients with chronic kidney disease patients do have routine monitoring in their guidelines (56?). The case is made for class 1A indication for beta-blocker use in patients with heart failure with reduced ejection fraction even when the heart rate is not elevated (26–27). In that situation, heart rate is a surrogate and possibly imperfect marker for upregulation of sympathetic nervous system in heart failure. In our small study, we found that monitoring 25(OH)D and subsequent repletion may have clinical merit with regard to improved QOL and neurohormonal indices. Determining if supplementation of vitamin D in all CHF patients without regard to baseline status will require further research.

### Inflammation

Vitamin D3 in our study did not appear to have a strong impact on hsCRP as a whole, although men had an almost 2 mg/L reduction at 6 months. A prior study showed that vitamin D3 given at 2000 IU per day for nine months in heart failure improved cytokine profiles [38], and both vitamin D and inflammation are related in heart failure [55]. Vitamin D impacts several inflammatory pathways and acts to inhibit the activity of nuclear factor B activity, and by reducing interleukin 6, interleukin 12, interferon C, and tumor necrosis factor alpha production [38]. A recent review of vitamin D and inflammation suggests that vitamin D possibly modestly improves inflammation overall and that it may have more anti-inflammatory effect with greater levels of inflammation in diseases such as end stage renal disease, lupus, and tuberculosis, but also has effects on inflammation in acute coronary syndrome [58]. Additionally, inflammation related to vitamin D defiency is associated with poor HF outcomes [59]. Both of our treatment arms were equally matched in regard to statin usage at baseline and had been on stable doses for at least 3 months, so this likely did not impact hsCRP in this study. A recent review of inflammation and vitamin D found that 6 out of 7 randomized clinical trials supplementing vitamin D found reductions in inflammatory markers [60]. Our research was exploratory in nature and was not powered to detect differences in hsCRP. Future studies with marked elevation in inflammation levels at baseline may be useful to further understand the role of vitamin D3 for inflammation in HF.

## Conclusions

Our study suggests that daily vitamin D3, used to replete insufficient stores, may safely improve hormonal factors in heart failure patients like 25(OH)D, BNP, and PTH as well as inflammation. In addition to independent hormonal modulation, sufficient vitamin D3 repletion may provide an important improvement in QOL as an adjuvant treatment to appropriate guideline directed therapy. Future considerations for research should additionally measure 1,25(OH)2D levels and determine if 25(OH)D- guided dosing is superior. Further work is needed to determine if a provision of nutrient cofactors further enhance the effectiveness of vitamin D.

## Additional files

Additional file 1: Data repository file. This file contains data for all elements used in this research study. (XLS 62 kb)

Additional file 2: CONSORT checklist. (PPTX 2673 kb)

## Abbreviations

1,25(OH)2D: 1,25-dihydroxyvitamin D
25(OH)D: 25-hydroxyvitamin D
ACE: Angiotensin Converting Enzyme
ARB: Angiotensin Receptor Blocker
BMI: Body Mass Index
BNP: B-type Natriuretic Peptide
BP: Blood Pressure
BUN: Blood Urea Nitrogen
CI: Confidence Interval
CO_2_: Carbon dioxide
CPX: Cardiopulmonary status
CRP: C-Reactive Protein
EF: Ejection Fraction
EGR1: Early Growth Response
HF: Heart Failure
HR: Heart Rate
hsCRP: High Sensitivity C-reactive Protein
IU: International Units
KCCQ: Kansas City Cardiomyopathy Questionnaire
LCZ696: Angiotensin-Neprilysin
METS: Metabolic Equivalents
NHANES: National Health and Nutrition Survey
NYHA: New York Heart Association
OUES: Oxygen Uptake Efficiency Slope
PETCO_2_: Partial Pressure End Tidal Carbon Dioxide
PETO_2_: Partial Pressure End Tidal Oxygen
PTH: Parathyroid Hormone
QOL: Quality of Life
RER: Respiratory Exchange Ratio
SD: Standard Deviation
TRIM27: Tripartite Motif Containing 27
V0_2_: Oxygen Production
VCO_2_: Oxygen consumption
Vol: Volume
