# Supplementary figures and images for: Vitamin D3 repletion versus placebo as adjunctive treatment of heart failure patient quality of life and hormonal indices: a randomized, double-blind, placebo-controlled trial
Source: BMC Cardiovasc Disord. 2017 Oct 30;17:274. doi: 10.1186/s12872-017-0707-y (PMC5663043; doi:10.1186/s12872-017-0707-y)

## Slide 1
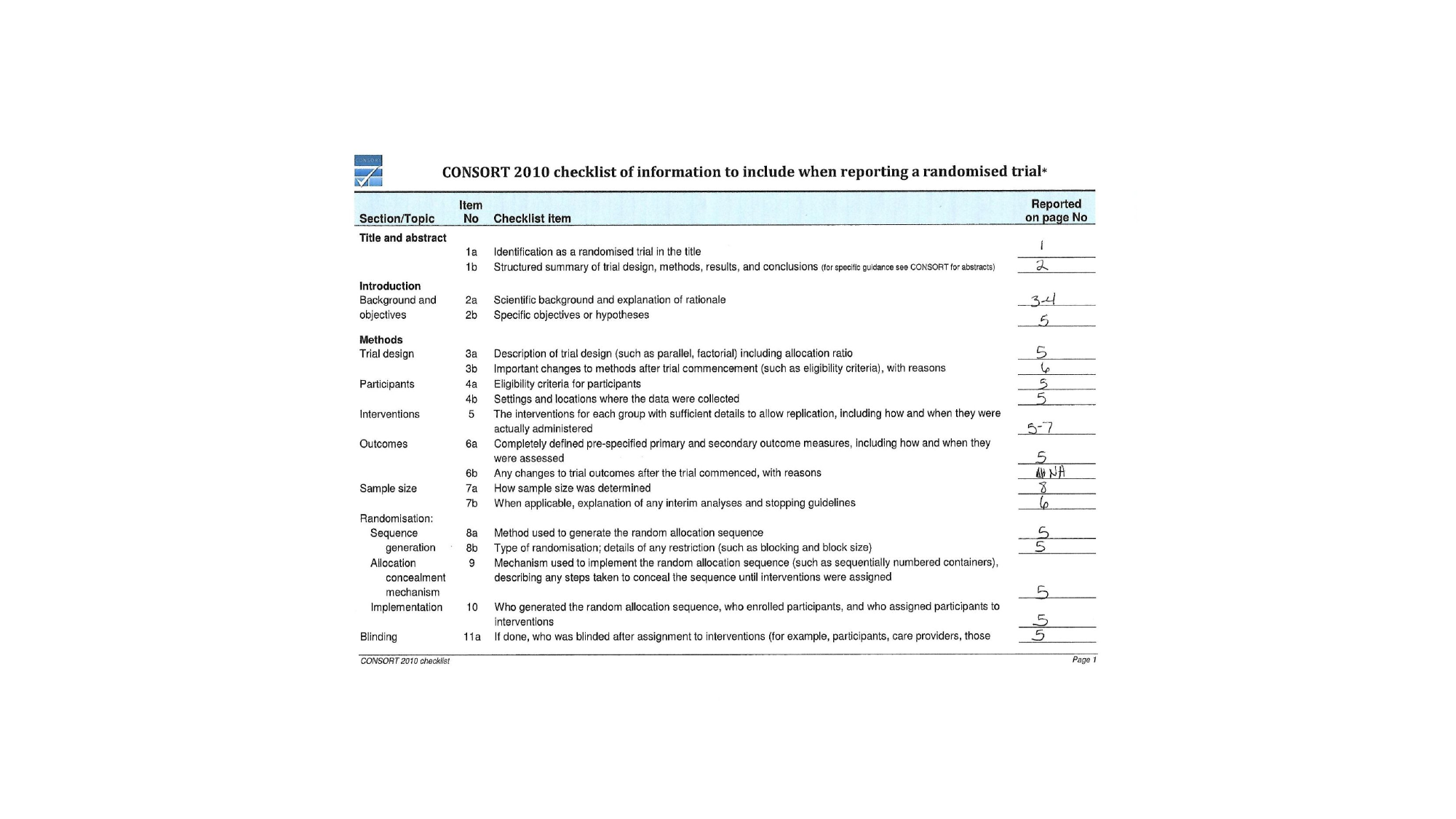

## Slide 2
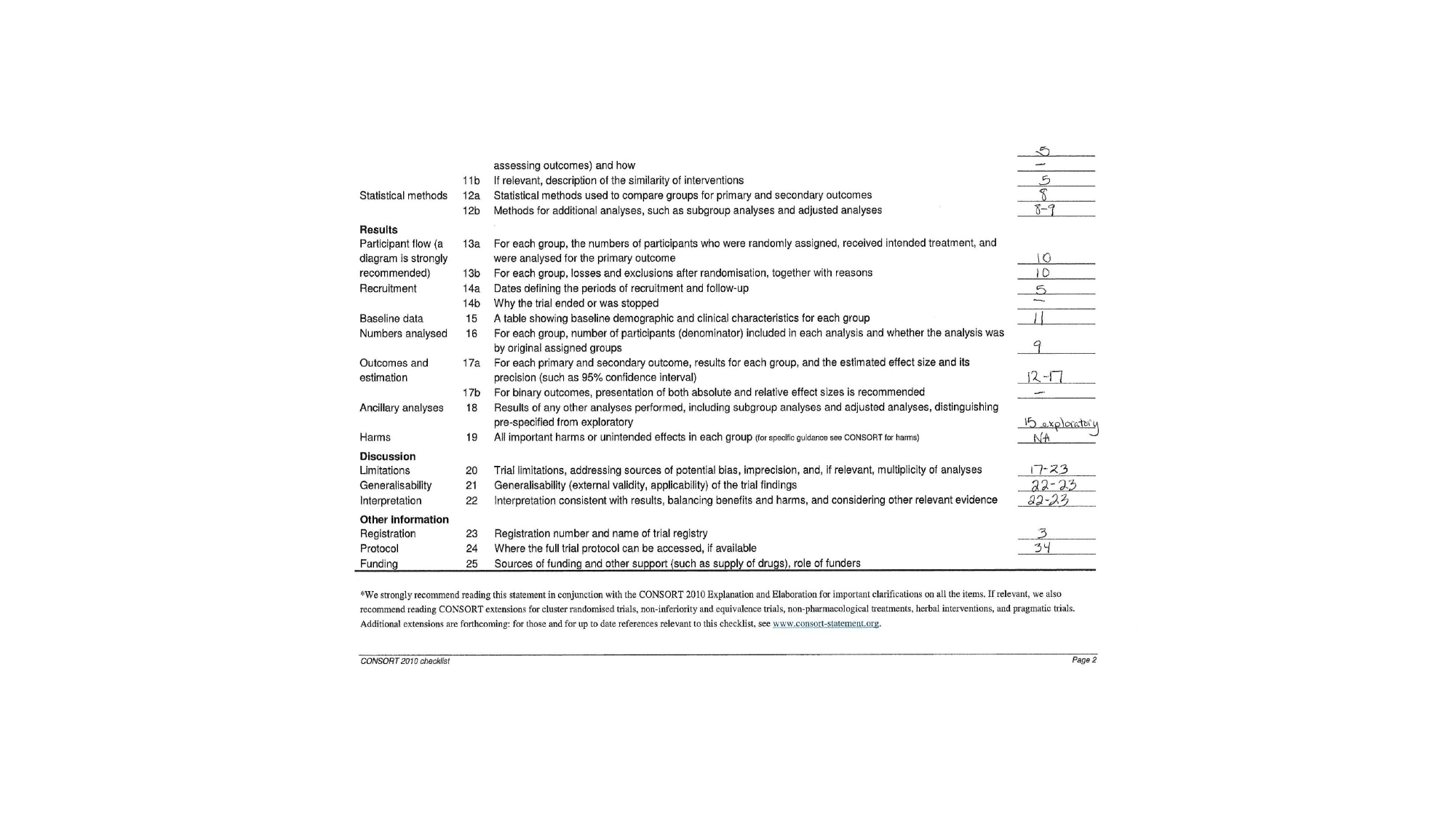

Supplement: Supplementary file 2 — CONSORT checklist. (PPTX 2673 kb) [file 12872_2017_707_MOESM2_ESM.pptx]
